# Large-scale implementation of standardized quantitative real-time PCR fecal source identification procedures in the Tillamook Bay Watershed

**DOI:** 10.1371/journal.pone.0216827

**Published:** 2019-06-06

**Authors:** Xiang Li, Mano Sivaganesan, Catherine A. Kelty, Amity Zimmer-Faust, Pat Clinton, Jay R. Reichman, York Johnson, William Matthews, Stephanie Bailey, Orin C. Shanks

**Affiliations:** 1 Oak Ridge Institute for Science and Education, Oak Ridge, TN, United States of America; 2 U.S. Environmental Protection Agency, Office of Research and Development, Cincinnati, OH, United States of America; 3 Southern California Coastal Water Research Project, Costa Mesa, CA, United States of America; 4 U.S. Environmental Protection Agency, Office of Research and Development, Newport, OR, United States of America; 5 U.S. Environmental Protection Agency, Office of Research and Development, Corvallis, OR, United States of America; 6 Oregon Department of Environmental Quality & Tillamook Estuaries Partnership, Garibaldi, Oregon, United States of America; 7 Oregon Department of Agriculture, Salem, Oregon, United States of America; 8 U.S. Environmental Protection Agency, Region 10 Manchester Laboratory, Port Orchard, WA, United States of America; University of Minnesota Twin Cities, UNITED STATES

## Abstract

Fecal pollution management remains one of the biggest challenges for water quality authorities worldwide. Advanced fecal pollution source identification technologies are now available that can provide quantitative information from many animal groups. As public interest in these methodologies grows, it is vital to use standardized procedures with clearly defined data acceptance metrics and conduct field studies demonstrating the use of these techniques to help resolve real-world water quality challenges. Here we apply recently standardized human-associated qPCR methods with custom data acceptance metrics (HF183/BacR287 and HumM2), along with established procedures for ruminant (Rum2Bac), cattle (CowM2 and CowM3), canine (DG3 and DG37), and avian (GFD) fecal pollution sources to (i) demonstrate the feasibility of implementing standardized qPCR procedures in a large-scale field study, and (ii) characterize trends in fecal pollution sources in the research area. A total of 602 water samples were collected over a one-year period at 29 sites along the Trask, Kilchis, and Tillamook rivers and tributaries in the Tillamook Bay Watershed (OR, USA). Host-associated qPCR results were combined with high-resolution geographic information system (GIS) land use and general indicator bacteria (*E*. *coli*) measurements to elucidate water quality fecal pollution trends. Results demonstrate the feasibility of implementing standardized fecal source identification qPCR methods with established data acceptance metrics in a large-scale field study leading to new investigative leads suggesting that elevated *E*. *coli* levels may be linked to specific pollution sources and land use activities in the Tillamook Bay Watershed.

## Introduction

Fecal pollution management represents a major challenge for water quality authorities worldwide. Fecal waste can enter waterways from a variety of sources such as leaky sewer lines, faulty septic systems, stormwater run-off, improper agricultural waste management practices, and local wildlife [1–5]. When fecal pollution is present in surface waters, it can represent a public health risk due to the presence of disease causing pathogens (see [6] for review), as well as lead to severe economic burdens [7]. Water quality managers employ general fecal indicator bacteria such as *E*. *coli* or enterococci for routine monitoring to identify locations with unsafe levels of fecal pollution [8]. These indicator bacteria are found in the feces of most animals and alert managers to the total pollution level, however, they do not provide any information about the cause or source of pollutants. The absence of fecal pollution source data limits the ability of a water quality manager to implement focused, cost-effective mitigation strategies, especially in catchments impacted by multiple animal groups.

Advanced fecal pollution monitoring technologies are now available that can provide quantitative source information from animal groups such as human [9–13], ruminant [9,14,15], cattle [16], canine [17–19], and avian [18,20] sources. As management and regulatory interest in these methodologies grows, it becomes vital to establish standardized procedures with clearly defined data acceptance metrics and conduct field studies demonstrating the use of these techniques to help resolve real-world water quality challenges. A recent multiple laboratory validation study proposed the first standardized human-associated qPCR procedures including tailored data acceptance metrics for environmental surface water sample testing with HF183/BacR287 and HumM2 methods [21] leading the recent public release of standardized methods by the United States Environmental Protection Agency [21–23]. However, to date, there are no large-scale implementation studies demonstrating the feasibility and utility of these new laboratory practices and data acceptance metrics. Field studies allow researchers to evaluate the performance of protocols and fine-tune strategies for future water quality management implementation ranging from site selection to data interpretation. As a greater number of communities face chronic water quality impairment challenges due to fecal pollution and qPCR technologies become more accessible, water quality managers are turning to these methods not only to remediate polluted sites, but to take steps to prevent future contamination on a source by source basis.

To evaluate the use of new qPCR fecal source identification protocols and data acceptance metrics, we conducted a large-scale field study in the Tillamook Bay Watershed with the following objectives: (i) implement recently reported standardized protocol and data acceptance metrics for the HF183/BacR287 and HumM2 human-associated qPCR methods in a large-scale field demonstration [22–24], and (ii) characterize fecal pollution trends in the study area. Surface water samples (n = 602) were collected over a one-year period from 29 sites along the Trask, Kilchis, and Tillamook tributaries (Tillamook Bay Watershed, OR) and subject to host-associated qPCR testing for human (HF183/BacR287 [23,24] and HumM2 [22,25]), ruminant (Rum2Bac [14]), cattle (CowM2 and CowM3 [16]), canine (DG3 and DG37 [17]), and avian (GFD [26]) fecal pollution. Host-associated qPCR fecal pollution results were combined with high resolution geographic information system (GIS) land use data, general fecal indicator bacteria measurements (*E*. *coli*), and local weather information to elucidate fecal pollution trends in the Tillamook Bay Watershed. Results clearly show that standardized human-associated HF183/BacR287 and HumM2 qPCR method implementation is feasible in a large-scale field study scenario. Fecal source identification findings also revealed evident human, ruminant, cattle, canine, and avian fecal pollution trends in the Tillamook Bay Watershed providing local public health authorities with new investigative leads to improve water quality management.

## Materials and methods

### Site description

**Fig 1:**
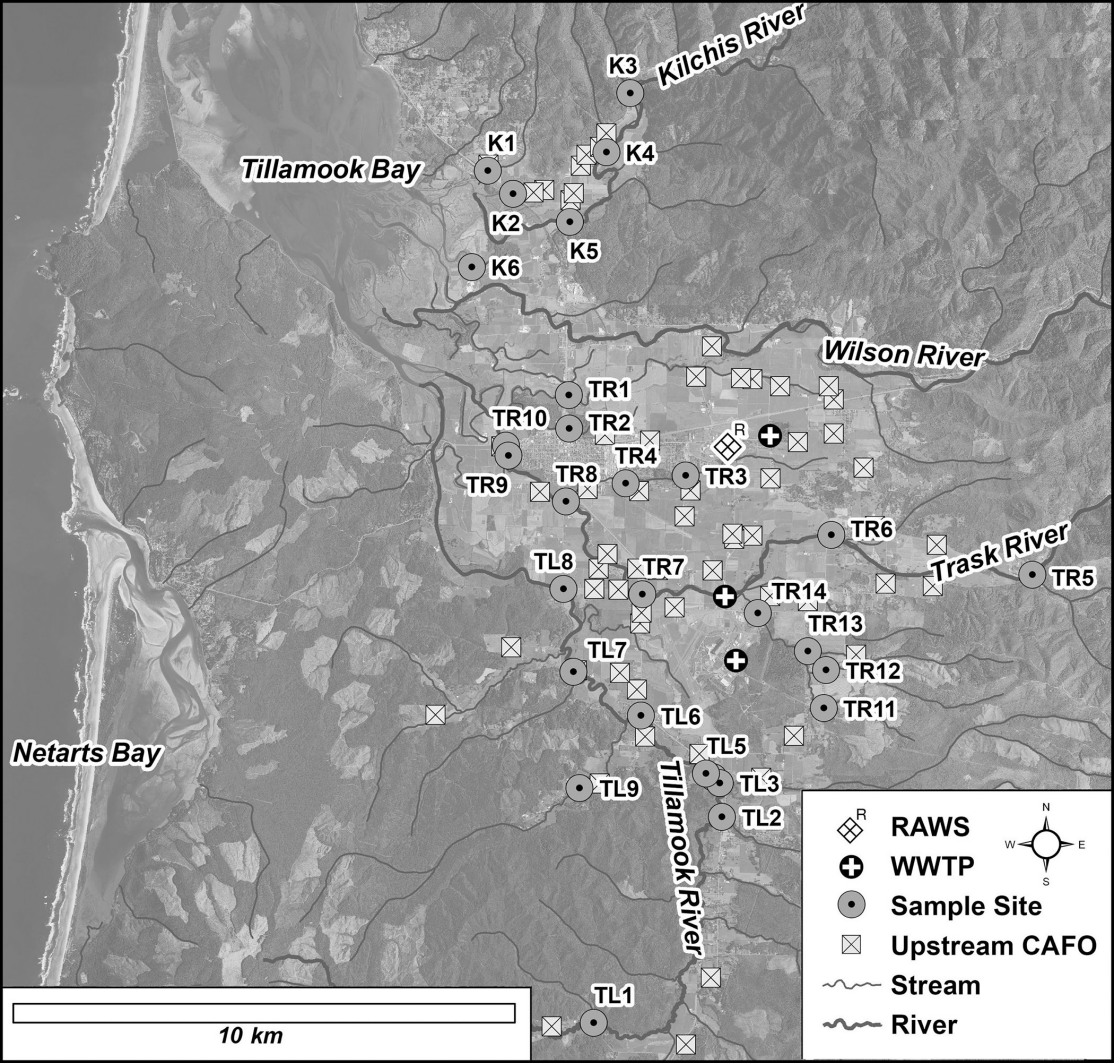
Global information system map of the Tillamook Bay Watershed study area showing locations of sampling sites, waste water treatment plants (WWTP), cattle confined animal feeding operation (CAFO) facilities, and weather station (RAWS).

The Tillamook Bay Watershed is a 1,500 km^2^ area situated along the northern Oregon coast consisting of agricultural lands, vast forested areas, and residential communities. The Tillamook Bay Watershed plays a vital role in supporting a thriving shellfish, dairy, and cheese-making industry, as well as providing a safe water resource for local human and wildlife populations. Fecal pollution is currently monitored in this region with standard *E*. *coli* cultivation methods [27] and counts greater than 406 organisms/100 mL from a single sample are considered unsafe for recreational use [28]. Twenty-nine sampling sites (Fig 1) from three major river tributaries [Tillamook (n = 9; TL1-TL9); Trask (n = 14; TR1-TR14); and Kilchis (n = 6; K1-K6)] were selected for water quality testing based on recommendations and permission from the Oregon Department of Environmental Quality. Site historical *E*. *coli* data collected over the past 10 years indicated a broad range of water quality conditions with exceedance probabilities (single-day maximum exceedance = 406 MPN/100mL) spanning from consistently acceptable water quality 0.4 ± 0.4% (K3 and K4) to chronically poor 77.5 ± 2.7% (TR11) (S1 Table). In addition, approximately 80% of sites (n = 23) exhibited no change in exceedance probabilities (p ≥ 0.01) suggesting that local management activities over the past decade were unable to improve water quality at many sites relying on *E*. *coli* fecal pollution characterization alone (see Supplemental Information for full historical *E*. *coli* analysis description). Fecal bacteria may enter these river sites from the 63 concentrated-animal-feeding-operations (CAFO), numerous smaller agricultural animal facilities, wastewater treatment plants (WWTP), public campgrounds, stormwater run-off, rural onsite septic systems, and indigenous wildlife. Known point sources of fecal waste are found on the Trask River (mobile home park and Industrial Park Sewage Treatment Plant) and Tillamook River (Port of Tillamook WWTP and City of Tillamook WWTP).

### Sample collection and *E*. *coli* enumeration

A total of 602 water samples were collected on a bimonthly schedule over a one-year study period (start date: July 2014). Samples were collected in sterile 1 L containers from surface water and were immediately stored on ice during transport to the laboratory. Culture-based enumeration of *E*. *coli* was performed with Colilert IDEXX defined substrate procedure as described by the manufacturer (IDEXX Laboratories, Inc. Westbrook, ME) within six hours of sample collection. For 598 samples (4 samples not eligible for DNA testing due to prolonged holding time > 8 h), 100 mL was filtered through a 0.45 μm polycarbonate filter (Fisher Scientific, Pittsburg, PA) within 8 hours of sample collection. Filters were placed in sterile 2 mL screw cap tubes containing a silica bead mill matrix (GeneRite, North Brunswick, NJ) and stored at -80°C (< 18 months) until DNA purification.

### Fecal pollution reference sample collection

A total of 109 reference fecal and four untreated sewage samples were collected from the Tillamook Bay Watershed region as previously described [29]. Fecal samples represent eight animal groups frequently found in the study area including adult cattle (n = 32; *Bos taurus*), juvenile cattle (n = 20; < 115 days old; *Bos taurus*), canine (n = 11; *Canis familiaris*), elk (n = 11; *Cervus elaphus*), horse (n = 11; *Equus caballus*), chicken (n = 11; *Gallus gallus*), gull (n = 3; Laridae), and Canada goose (n = 10; *Anser* sp.). Each fecal sample was collected from a different individual. Single grab, untreated sewage samples were collected from the City of Tillamook WWTP (n = 2) and Industrial Sewage Treatment Plant (n = 2). Briefly, 25 mL of sewage was filtered through a 0.45 μm polycarbonate filter (Fisher Scientific, Pittsburg, PA) within 8 hours of sample collection. Filters were placed in sterile 2 mL screw cap tubes containing a silica bead mill matrix (GeneRite, North Brunswick, NJ) and stored at -80°C (< 18 months) until DNA purification.

### Sample site land use and weather data collection

Geographic information system (GIS) mapping was used to define sample site catchment drainage areas and generate land use datasets using ArcGIS ArcMap (Version 10.2.2; ESRI, Redlands, CA). Site catchment drainage areas were defined with the Spatial Analysis Hydrology tool using stream and elevation data from the National Hydrology Dataset (http://datagateway.nrcs.usda.gov). The human population in each catchment area was estimated using the EnviroAtlas Dasymetric toolbox [30]. Pacific Northwest Spatially Referenced Regression on Watershed Attributes (SPARROW) data layers were used to estimate the maximum permitted CAFO cattle population (total count/catchment area; Oregon Spatial Data Library http://spatialdata.oregonexplorer.info/geoportal) and percent non-sewer area (% of catchment area) datasets [31]. Percent of cropland in a catchment area was calculated from the National Gap Analysis Project Land Cover dataset (https://gapanalysis.usgs.gov/gaplandcover/). Cumulative rainfall (mm per 120, 72, and 24 h prior to sampling event), solar irradiance (kW-hr/m^2^; 24 h prior to sampling event) and air temperature (°C; 24 h prior to sampling event) were calculated from the Western Regional Climate Center for Tillamook, OR (https://wrcc.dri.edu/).

### Reference DNA preparation

Reference DNA sources consisted of two plasmid constructs (Integrated DNA Technologies, Coralville, IA) and salmon sperm DNA (Sigma-Aldrich, St. Louis, MO. Plasmid constructs for calibration standards (all targets on single construct) and internal amplification controls (IAC) were linearized by Not1 restriction digest (New England BioLabs, Beverly, MA), purified via QIAquick PCR Purification kit (Qiagen, Valencia, CA), quantified with Quant-it PicoGreen ds DNA Assay Kit (Thermo Fisher Scientific, Grand Island, NY) on a SpectraMax Paradigm Multi-Mode Microplate Detection Platform (Molecular Devices, Sunnyvale, CA), and diluted in 10 mM Tris 0.1 mM EDTA (pH 8.0) to generate 10, 10^2^, 10^3^, 10^4^, 10^5^ copies/2μl for calibration standards and 10^2^ copies/2μL for IAC reference material. A salmon DNA working stock containing 10 μg/mL was prepared by diluting the commercially available 10 mg/mL solution. All reference DNA material preparations were stored in GeneMate Slick low-adhesion microcentrifuge tubes (ISC BioExpress, Kaysville, UT) at -20°C.

### DNA purification

For water samples, 600 μL of 0.02 μg/mL salmon sperm DNA (Sigma-Aldrich, St. Louis, MO) was added to each bead mill tube prior to DNA purification. For each reference fecal sample, a fecal slurry was prepared consisting of molecular grade PBS and fecal matter (~1:1 ratio). Approximately 1 mL of fecal slurry was transferred to a sterile 2 mL screw cap tube containing a silica bead mill matrix (GeneRite, North Brunswick, NJ). Bead milling was achieved with a MP FastPrep-24 (MP Biomedicals, LLC Solon, OH) at 6.0 m/s for 30 s. DNA purification was performed using the DNA-EZ kit (GeneRite, North Brunswick, NJ) according to manufacturer’s instructions. Three method extraction blanks (MEB), with purified water substituted for test sample, were performed with each sample processing batch (38 samples/batch). DNA was eluted with 100 μL elution buffer into GeneMate Slick low-adhesion microcentrifuge tubes (ISC BioExpress, Kaysville, UT). For water filter samples, DNA extracts were stored at 4°C prior to qPCR amplification (< 48 h). For sewage and fecal DNA samples, DNA extraction yields were determined with a NanoDrop ND-1000 UV spectrophotometer (NanoDrop Technologies, Wilmington, DE), diluted to a test concentration of 0.5 ng/μL in 10 mM Tris 0.1 mM EDTA (pH 8.0), and stored at -20°C until amplification (< 6 months).

### qPCR amplification

Nine qPCR assays were used in this study, including two human-associated assays (HF183/BacR287 and HumM2), two cow-associated assays (CowM2 and CowM3), a ruminant-associated assay (Rum2Bac), two canine -associated assays (DG3 and DG37), an avian-associated assay (GFD), and a sample processing control (SPC) assay (Sketa22) as previously reported (refer to S2 Table for more details) with the following modifications [14,16,17,22–26,32]. Reaction mixtures contained 1X TaqMan Environmental Master Mix (version 2.0; Thermo Fisher Scientific, Grand Island, NY), 0.1X SYBR Green I Dye (GFD assay only; Thermo Fisher Scientific, Grand Island, NY), 0.2 mg/mL bovine serum albumin (Sigma-Aldrich, St. Louis, MO), 1 μM each primer, and 80 nM 6-carboxyfluorescein (FAM)-labeled probe, and 80 nM VIC-labeled probe (multiplex reactions only). All reactions contained either 10 to 1x10^5^ target gene copies of reference DNA calibration standard or 2 μL of DNA sample extract in a total reaction volume of 25 μL. Multiplex reactions with HF183/BacR287 and HumM2 also contained 10^2^ copies of IAC template. All reactions were performed in triplicate in MicroAmp optical 96-well reaction plates with MicroAmp 96-well optical adhesive film (Thermo Fisher Scientific, Grand Island, NY). The thermal cycling profile for all assays was 2 min at 95°C followed by 40 cycles of 5 s at 95°C, and 30 s at 60°C (except GFD, 57°C). The threshold was manually set to either 0.03 (HF183/BacR287, DG3, DG37 and Sketa22) or 0.08 (HumM2, CowM2, CowM3 and GFD), and quantification cycle (Cq) values were exported to Microsoft Excel for further data analysis. To monitor for potential extraneous DNA contamination during qPCR amplification, six no-template controls (NTC) with purified water substituted for template DNA were performed with each instrument run.

### Data acceptance metrics

A series of acceptance metrics designed to ensure high quality data generation were used in this study [21]. Briefly, HF183/BacR287 and HumM2 multiplex IAC procedures were used to monitor for amplification inhibition. Any DNA extract indicating evidence of amplification inhibition was discarded. A SPC protocol was used to identify suitable DNA recovery from each water sample. Water samples with unacceptable DNA recovery were excluded from the study based on batch-specific (n = 38 samples/batch) criteria derived from repeated method blank spike recovery measurements. SPC proficiency was also assessed for each water sample batch preparation requiring a standard deviation in Sketa22 qPCR method extraction repeated measures of ≤ 0.62 Cq. In addition, HF183/BacR287 and HumM2 were subject to calibration model [linearity (R^2^ ≥ 0.980)] and amplification efficiency (0.90 to 1.10 where *E* = 10^(-1/slope)^– 1) acceptance criteria, as well as instrument run-specific IAC proficiency testing (HF183/BacR287 and HumM2 NTC VIC Cq standard deviation ≤ 1.16 or 1.05, respectively).

### Data analyses

Master calibration models were generated for each qPCR assay from six independent standard curves using a Bayesian Markov Chain Monte Carlo approach [33]. The lower limit of quantification (LLOQ) was defined as the 95% credible interval upper bound from repeated measurements (n = 18) of 10 copies per reaction reference DNA standard dilutions. qPCR target concentration estimates were reported as mean log_10_ copy number per reaction. Sensitivity was defined as the total number of correct positive reactions divided by the total number of reactions containing the target pollution source (sensitivity = TP/(FN + TP), where TP and FN are true positives and false negatives, respectively). Specificity was calculated as the total number of correctly identified negative reactions divided the total number of reaction that do not contain the target pollution source (specificity = TN/(FP + TN) where TN and FP are true negatives and false positives, respectively). To investigate potential land use and weather trends in qPCR measurements, average log_10_ copies per reaction were estimated using a maximum likelihood estimation method by either sampling site (land use) or sampling day (weather). Average log_10_ MPN/100mL values were used for *E*. *coli* measurements. Water quality metrics were considered eligible for trend analysis if more than 20% of respective average concentrations (sampling site or sampling day) were greater than zero. For weather trend analysis, eligible sampling day log_10_ copies per reaction concentrations were binned into two groups based on paired precipitation, solar irradiance, or air temperature median values (Table 1) and subject to a non-parametric Mann Whitney U test (α = 0.05). For land use trend analysis, eligible sampling site log_10_ concentrations and paired human population, maximum permitted CAFO cattle population, percent non-sewer area, and percent cropland measurements were subject to multiple linear regression (α = 0.05). Sample sites were ranked for each qPCR assay using a weighted average fecal score utilizing all measurements including non-detects as reported elsewhere [34]. R statistical package (version 3.1.1) was used to generate heat maps (gplots) and to perform Firth’s logistic regression analyses (brglm). All statistics were calculated with SAS software (Cary, NC) and Microsoft Excel.

**Table 1:** Land use and weather condition summary statistics over study period.

Type	Parameter	Mean	Median	Std.	Min.	Max.
Land Use	Human Population (number per catchment area)	1051.6	533	1727.1	2	6395
	Maximum Permitted CAFO Cattle Population (number per catchment area)	2418.9	826	3472.7	0	12,371
	Non-sewer (% of catchment area)	1.06	0.18	1.83	0.006	7.44
	Cropland (% of catchment area)	1.97	0.53	4.89	0.08	25.7
Weather Condition	120-h precipitation (mm)	27.3	9.5	36.6	0	168.9
	72-h precipitation (mm)	16.4	3.8	25.9	0	144.5
	24-h precipitation (mm)	5.5	0.1	12.3	0	101.1
	Solar Irradiance (kW-hr/m^2^)	3.5	2.9	2.3	0.1	8
	Air Temperature (°C)	11.9	12	3.6	0.7	23.9

Human population estimated using EnviroAtlas Dasymetric toolbox [30].

Maximum permitted CAFO cattle population and percent non-sewered area estimated using SPARROW data layers [31].

Percent cropland estimated from the National Gap Analysis Project Land Cover data set (https://gapanalysis.usgs.gov/gaplandcover/).

Cumulative rainfall, solar irradiance, and air temperature prior to sampling calculated from Western Regional Climate Center for Tillamook, OR data sets (https://wrcc.dri.edu/).

## Results and discussion

### qPCR quality controls and data acceptance metrics

All qPCR experiments were subject to a rigorous series of quality controls and data acceptance metrics to ensure the use of high-quality data for fecal source identification. Calibration model performance parameters and IAC thresholds for each qPCR assay are shown in S3 Table. Calibration model R^2^ values were all greater than 0.981, and *E* values ranged from 0.90 (GFD) to 0.97 (HF183/BacR287). Of the 598 water filters, six DNA extracts (1.0%) were discarded from the study due to severe matrix interference based on SPC tests. SPC acceptance thresholds ranged from 21.9 C_q_ to 25.5 C_q_. A total of 217 filter DNA extracts were eligible for C_q_ adjustments ranging from 0.004 to 3.09. Acceptable DNA recovery was monitored for each extraction batch (n = 38 samples/batch) using the SPC proficiency test [21]. Sketa22 MEB Cq values ranged from 21.9 to 25.5 with standard deviations from 0.11 to 0.85 across 26 batch preparations resulting in a successful SPC proficiency rate of 92.3% (24 of 26 batches). The two batches that failed the SPC test (MEB Sketa22 Cq standard deviation ≤ 0.62) were discarded from the study. Instrument run-specific IAC proficiency testing yielded a 100% pass rate with NTC VIC Cq standard deviations ranging from 0.20 to 0.82 for HF183/BacR287 (acceptance criteria ≤ 1.16) and 0.17 to 0.87 for HumM2 (acceptance criteria ≤ 1.05). Amplification inhibition was rarely identified in multiplex IAC HF183/BacR287 and HumM2 experiments [1.12%; 8 of 713 total DNA extractions (fecal and filter)]. IAC acceptance thresholds ranged from 32.8 C_q_ to 37.8 C_q_ (HF183/BacR2876) and 34.4 C_q_ to 38.6 C_q_ (HumM2). Competition thresholds were 27.9 C_q_ for HF183/BacR287 and 30.1 C_q_ for HumM2. Extraneous DNA control reactions indicated 99.95% DNA-free (2 positives of 4,200 total reactions). False positives were both from HF183/BacR287 tests (39.2 C_q_ from a MEB; 37.1 C_q_ from a NTC).

### Implementation of HF183/BacR287 and HumM2 qPCR standardized methods

The standardization of fecal source identification qPCR laboratory practices and development of data acceptance criteria are critical for these technologies to transition from a subject of environmental microbiology research to useful water quality management and safety planning tools. In 2016, a team of researchers published the first standardized qPCR methodology including custom data acceptance metrics for two top performing human-associated fecal source identification technologies [21], which were incorporated into recently released United States Environmental Protection Agency draft methods [21–23]. Recommended practices were adapted from the essential minimum information for publication of qPCR experiments (MIQE) guidelines [35] and a multiple laboratory validation study [21] to promote experimental transparency, help ensure consistency between laboratories, and enhance the integrity of these fecal source identification methods. Although there are clear advantages to standardized procedures and data acceptance metrics, there are currently no field studies demonstrating the implementation of these practices. Here, we report the first large-scale field application of these proposed standardized protocols and data acceptance criteria for the United States Environmental Protection Agency HF183/BacR287 and HumM2 qPCR draft methods [21–23].

Systematic surveillance of standardized HF183/BacR287 and HumM2 quality control and data acceptance data from the Tillamook Bay Watershed field study revealed two important observations. First, it is feasible to employ all proposed data acceptance metrics in a large-scale study. Notably, proficiency tests specifically designed to ensure proper implementation of DNA recovery (SPC) and amplification inhibition (IAC) controls demonstrated acceptable performance in 92% (DNA recovery) and 100% (amplification inhibition) of experiments. Second, poor DNA recovery and amplification inhibition were absent in more than 98% of water samples tested suggesting that custom environmental reagents and standardized DNA purification practices can consistently yield suitable DNA for genetic testing in environmental conditions. It is important to note that this case study focused on freshwater samples collected from rivers in the Tillamook Bay Watershed. Future large-scale field research studies are warranted to assess the performance of these technologies across a broader range of geographic locations and water types (i.e. marine).

### Fecal pollution trends in the Tillamook Bay Watershed

**Fig 2:**
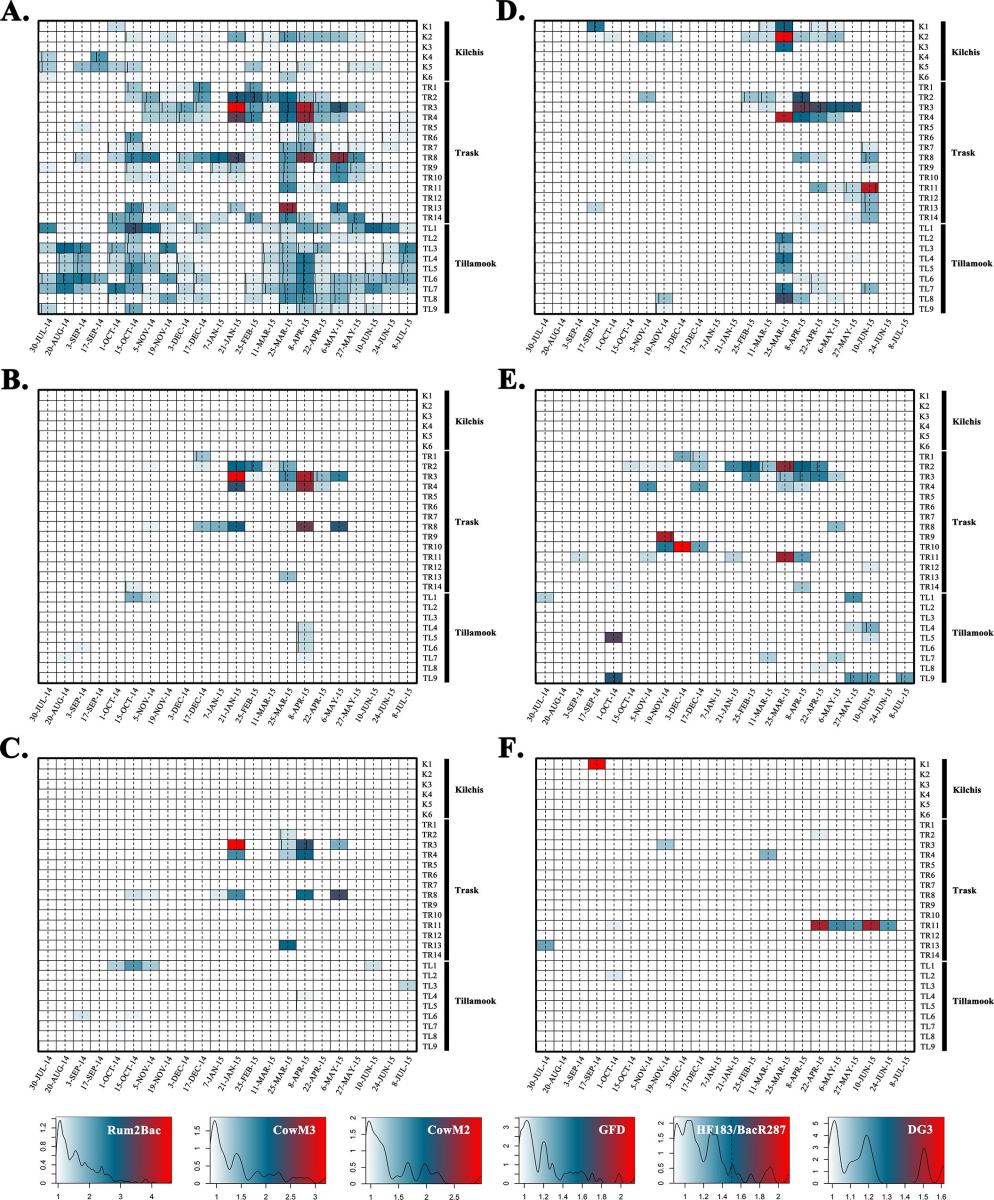
Heat map illustrating measurements of host-associated qPCR genetic marker estimated log_10_ copies per reaction concentrations for Rum2Bac (Panel A), CowM3 (Panel B), CowM2 (Panel C), GFD (Panel D), HF183/BacR287 (Panel E), and DG3 (Panel F). Estimated concentrations are organized by sampling event time (y-axis) and sampling site (x-axis). Heat map keys are shown at bottom reporting estimated log_10_ copies per reaction color coding and frequency information.

Fecal source identification qPCR methods for human (HF183/BacR287 and HumM2), ruminant (Rum2Bac), cattle (CowM2 and CowM3), canine (DG3 and DG37), and avian (GFD) were combined with *E*. *coli* cultivation measurements to characterize fecal pollution in water samples collected from the Tillamook Bay Watershed. Estimated mean log_10_ copies per reaction concentrations for host-associated qPCR methods are shown in Fig 2 [data for HumM2 and DG37 not shown (< 1% in ROQ)]. *E*. *coli* site average log_10_ MPN/100mL concentrations ranged from 1.38 (K3) to 2.76 (TR11) (S4 Table). Table 2 shows the top five sampling sites with the highest average *E*. *coli* log_10_ MPN/100mL concentrations and corresponding rankings for each host-associated genetic marker (average log_10_ copies/reaction). For a complete list of site rankings, refer to S4 Table.

**Table 2:** Top five sites with highest average *E*. *coli* log_10_ MPN/100mL concentrations and respective fecal source identification site rankings by qPCR assay.

Site	*E*. *coli*^*€*^		Rum2Bac^¥^		CowM3^¥^		CowM2^¥^		HF183/BacR287^¥^		GFD^¥^		DG3^¥^	
	*Rank*	*Score*	*Rank*	*Score*	*Rank*	*Score*	*Rank*	*Score*	*Rank*	*Score*	*Rank*	*Score*	*Rank*	*Score*
TR11	1	2.76	NR	-0.04	NR	-0.47	NR	-1.08	9	0.14	9	0.04	1	0.12
TL1	2	2.52	5	1.79	NR	-0.27	NR	-0.15	NR	-0.26	8	0.07	NR	-0.85
TR3	3	2.46	1	2.12	1	1.02	1	0.35	4	0.22	2	0.46	NR	-0.70
TR8	4	2.39	6	1.58	3	0.33	NR	-0.04	NR	-0.34	4	0.31	NR	-1.12
TR4	5	2.38	2	1.87	2	0.50	NR	-0.20	2	0.44	7	0.20	NR	-0.20

^*€*^ Reporting unit for *E*. *coli* is log_10_ MPN/100mL

^¥^ Reporting unit is mean log_10_ copies/reaction

NR denotes ‘not ranked’

Water quality measurements were compared to land use and weather data to uncover potential trends in fecal pollution. GIS mapping was used to define catchment drainage boundaries allowing for the estimate of human population (total count/catchment area), non-sewer (% of catchment area), cropland (% of catchment area), and maximum permitted CAFO cattle population (total count/catchment area) associated with each sampling site (Table 1). Across the study area, human populations ranged from two (K6) to 6,395 (TR10) individuals, while permitted CAFO cattle populations ranged from zero (K6, K4, K3, K1, TR12, TR11, TR5, and TL9) to 12,371 (TR3). Site average concentrations were calculated for each eligible water quality metric to investigate potential correlations with land use parameters (S5 Table). Four water quality metrics were eligible for land use analysis including *E*. *coli* (100% of sampling site averages > 0), Rum2Bac (75.9%), GFD (34.5%), and HF183/BacR287 (24.1%). Descriptive statistics and daily measurements for air temperature (°C), solar irradiance (kW‧hr/m^2^), and precipitation (mm) are reported in Table 1 and S2 Fig, respectively. Solar irradiance was positively correlated with air temperature (r = +0.42, p < 0.001), but negatively with precipitation (r = -0.50, p < 0.001; r = -0.47, p < 0.001; and r = -0.44, p < 0.001 with 120-h, 72-h and 24-h precipitation definitions, respectively). On sampling days, the daily average air temperature ranged from 5.9 to 17.7°C, solar irradiance spanned 0.88 to 7.82 kW‧hr/m^2^, and no precipitation (per a 72-h accumulation period) was observed on 50% of days (11 of 22). Three water quality metrics were eligible for comparing potential links between water quality measurements and weather parameters including *E*. *coli* (100% of sampling day averages > 0), Rum2Bac (55.9%), and GFD (23.8%). Detailed findings are presented and discussed below organized by *E*. *coli*, ruminant, avian, human, and canine fecal pollution trends.

#### *E*. *coli* fecal pollution trends

Fecal pollution management in the Tillamook Bay Watershed is a year-round challenge. Using the local regulatory criteria for a single grab surface water sample (*E*. *coli* count ≥ 406 MPN/100mL), study sites were impaired from 0% (K3, K4, and K6) to 80% (TR11) of the time (S1 Fig). This broad range of water impairment suggests at least two different fecal pollution trends including (1) a chronic source of fecal pollution at some sites that is independent of local weather or time of sampling, and (2) episodic events linked to seasonal factors such as rainfall, agricultural practices, and/or wildlife activities. *E*. *coli* levels were positively correlated with 24-h precipitation, solar irradiance, and air temperature (p ≤ 0.003) further supporting a link between local weather and the occurrence of fecal pollution. In addition, a significant positive correlation between *E*. *coli* and percent non-sewered area (+R^2^ = 0.23; p = 0.0001) was observed suggesting that local septic system use may be a contributing factor. While *E*. *coli* testing confirms the presence of unsafe levels of fecal pollution at several sites and suggests rainfall and septic systems are contributing factors to poor water quality in the Tillamook Bay Watershed, these measurements do not specify pollution sources making it difficult to plan cost-effective remediation efforts.

#### Ruminant fecal pollution trends

The Tillamook Bay Watershed is home to a large dairy cattle population producing more than 300,000 tons of manure each year [36]. The ruminant-associated Rum2Bac was the most prevalent genetic marker found at measurable concentrations in 54.3% of water samples (Fig 2, Panel A) suggesting that the estimated 70,147 permitted CAFO cattle housed in the 63 CAFO facilities in the study area likely have a strong influence on local water quality conditions. However, the study area is also home to a much smaller number of elk (3,600 animals [37]), which are also ruminants and could be a possible source of the Rum2Bac genetic marker. While Rum2Bac does not discriminate between elk (*Cervus canadensis*) and cattle (*Bos taurus*), CowM2 and CowM3 genetic markers do and can therefore confirm the presence of cattle fecal pollution in a water sample. These methods identified the presence of measurable concentrations of cattle fecal pollution in 8.9% of samples successfully identifying this pollution source at 58.6% (17 of 29) of study sites (Fig 2, Panels B and C). The highest average Rum2Bac genetic marker concentration was found at the TR3 site (log_10_ 2.12 copies/reaction). This location also had the highest average concentration of cow-associated CowM2 (log_10_ 0.35 copies/reaction) and CowM3 (log_10_ 1.02 copies/reaction) genetic markers affirming the presence of cattle fecal pollution at this site. The TR3 catchment area includes three dairy cattle CAFO facilities permitted to house up to 2,205 individual animals. Across all study sites, Rum2Bac genetic marker concentrations were positively associated with the permitted number of CAFO cattle and precipitation (+R^2^ = 0.5; p = 0.0001). In addition, Rum2Bac concentrations were significantly correlated all weather conditions (p ≤ 0.002) suggesting a catchment area source loading potential consistent with rainfall run-off models, in which ruminant pollution builds up on the landscape between storm events and is washed off during subsequent rain storms. In addition, cattle roughly outnumber elk 20:1 in the study area and increases in Rum2Bac genetic marker concentrations were significantly correlated with the number of permitted CAFO cattle. However, it is still possible that elk also contribute to water quality challenges in the Tillamook Bay Watershed. To help identify sampling sites with a potentially high likelihood of elk fecal pollution impact, it will be necessary to obtain more accurate elk population and seasonal movement information or develop an elk-associated genetic marker for future water quality testing.

#### Avian fecal pollution trends

Fecal waste from bird species can harbor general fecal indicator bacteria such as *E*. *coli*, as well as a range of pathogens that can potentially infect humans and contribute to poor water quality [38–41]. The Tillamook Bay Watershed is home to several resident bird species, as well as numerous seasonal populations that migrate from the bay area into the river upper reaches during the early spring and summer that could impact local water quality (personal communication from Avian Predation Coordinator, Oregon Department of Fish & Wildlife). The GFD avian-associated genetic marker was used to reveal information about the potential influence of bird fecal waste in surface waters (Fig 2, Panel D). Overall, GFD was the second most frequently measured pollution source in water samples. Even though the GFD marker was measured in 13.1% of water samples, concentrations were not correlated with weather conditions (p ≥ 0.167) suggesting that bird fecal contamination is not primarily driven by a rainfall run-off mechanism unlike ruminant pollution. Instead, A Mann-Whitney rank sum test revealed that the GFD genetic marker concentration was significantly higher (p = 0.003) during a 10-week period (March 25, 2015 through June 10, 2015) compared to other sampling dates combined suggesting that a seasonal bird migration may influence local water quality. However, another possible explanation could be a seasonal change in the diet of resident birds triggering an increased shedding of the GFD marker during this time of year. Additional research is needed to further investigate the cause of a seasonal GFD genetic marker increase in the Tillamook Bay Watershed study area.

#### Human fecal pollution trends

There are an estimated 29,758 residents in the study area, roughly half the number of permitted CAFO cattle. Human waste can potentially enter local waters in the Tillamook Bay Watershed from wastewater treatment plants, public campgrounds, potential stormwater sewer cross-connections, faulty onsite septic systems, seasonal portable restrooms at local parks, and transient camps. In addition to containing *E*. *coli*, these human waste sources can harbor numerous pathogens (e.g., *Shigella sonnei*, noroviruses, and *Cryptosporidium* [42]), solids, debris, and a variety of pollutants (i.e., antibiotics, hormones, caffeine, steroids, metals, and synthetic organic compounds [43]). The incidence of human fecal pollution in the study area (Fig 2, Panel E) was much more erratic compared to ruminant and avian sources (Fig 2, Panels A and D, respectively), but a closer investigation reveals several interesting patterns. For example, measurable levels of the human-associated HF183/BacR287 genetic marker were observed on all but three sampling days at one or more site locations over the study period, however 48.3% (14 of 29) of sites exhibited no evidence of human fecal pollution (Fig 2, Panel E). In addition, each river tributary (Kilchis, Trask, and Tillamook) exhibited a different human fecal pollution temporal occurrence trend. Measurable levels of human fecal pollution in Trask River water samples typically occurred during the wet season (October 2014 through May 2015), while the opposite was true in Tillamook River samples. In contrast to a previous fecal source identification project conducted more than 10 years ago where the HF183 genetic marker was detected in 27% of Kilchis River samples [44], no measurable human fecal pollution was identified in Kilchis samples, regardless of sampling time suggesting water quality management efforts have reduced the incidence of human fecal contamination. River tributary-associated trends suggest that human fecal pollution in the Tillamook Bay Watershed is closely linked to adjacent land use activities and local waste management practices. This notion is supported by the positive association between percent non-sewer residential areas and HF183/BacR287 concentrations in the study area (+R^2^ = 0.32; p = 0.009).

#### Canine fecal pollution trends

Prior to this study, the extent to which dogs contribute to fecal pollution in the Tillamook Bay Watershed was unknown. While dog waste is thought to represent a lower public health risk compared to human and cattle sources [45], it does harbor *E*. *coli* [46] making it a potential contributor to surface water impairment. Evidence of dog contamination was sporadic, only occurring at a quantifiable concentration at eight sites and never on more than one consecutive sampling date, except for the TR11 site (Fig 2, Panel F). The TR11 site exhibited a consistently high concentration of the DG3 genetic marker between April 22, 2015 and June 24, 2015 clearly suggesting a water quality impact from pet waste management activities. The TR11 site was also impaired based on local criteria for *E*. *coli* 80% of the time (S1 Fig) including all instances when DG3 was detected at a measurable level (Fig 2, Panel F). A recent study reported an average *E*. *coli* concentration of 5.03 log_10_ colony forming units per milligram of wet fecal material in a collection of canine samples from California [46] suggesting this source of waste could contribute to water impairment at the TR11 site. The management of dog waste in the Tillamook Bay Watershed is left up to voluntary owner responsibility. Others report that that community education programs about good pet waste management practices can improve the water quality in areas impacted by canine fecal pollution [47].

### Implications for qPCR water quality data interpretation

The use of qPCR for fecal source identification is a promising technique offering valuable new insights to researchers and water quality managers. Even though standardizing qPCR laboratory practices and data acceptance criteria are critical for the widespread application of these technologies, interpreting qPCR results remains a major challenge due to numerous obstacles ranging from the potential for regional differences in method performance to selecting the appropriate statistical data analysis approach. The Tillamook Bay Watershed large-scale field study reported here provides an enormous data set offering important insights regarding quantitative fecal source identification data interpretation.

#### Performance evaluation with local fecal pollution reference sources

Assembly and testing of a local reference fecal pollution collection is a valuable strategy to help interpret host-associated qPCR data. In this study, a reference collection was systematically tested leading to important insights on the occurrence of host-associated genetic markers in the Tillamook Bay Watershed. First, specificity ranged from 99.1% (HumM2) to 100% (Rum2Bac, CowM3, GFD, DG3 and DG37) suggesting that if a host-associated genetic marker was observed in a water sample, the presence of the respective pollution source can be interpreted with a high degree of confidence. Second, Rum2Bac, CowM2, and CowM3 genetic markers were undetectable in all calf samples (< 115 days in age), but present in 36.5% (CowM2), 82.3% (CowM3), and 93% (Rum2Bac) of adult samples tested (> 6 months in age) indicating that these methods could underestimate the total cattle impact (juvenile + adults) in the study area. This same trend was reported in a dairy calf population from another agricultural facility (GA, U.S.A.) suggesting these ruminant-associated genetic markers may be absence in calves across the United States [48]. Third, Rum2Bac genetic marker concentration was 24 to 100 times higher compared to CowM2 and CowM3 genetic marker concentrations, respectively, in the same mass of fecal material (estimated log_10_ copies per ng of total DNA are shown in S3 Fig for target and non-target fecal pollution sources). As a result, when cattle waste is diluted over time in a local receiving water, the ability to identify CowM2 and CowM3 genetic markers will diminish before Rum2Bac. This has important ramifications for qPCR data interpretation suggesting that the absence of CowM2 and CowM3 combined with the presence of Rum2Bac does not necessarily implicate another ruminant pollution source such as elk in the Tillamook Bay Watershed. Another plausible explanation could be that the Rum2Bac originated from cattle, but the fecal waste has become too dilute to confirm with CowM2 and CowM3 genetic markers. Fourth, reference pollution testing with canine -associated methods showed that sensitivity of the DG3 (97%) was superior to DG32 (36.4%), suggesting that DG3 will be more successful for fecal source identification of dog waste in Tillamook waters. In contrast, another study reported that DG37 (85%) sensitivity was higher than DG3 (77%) in reference samples collected from four geographically separated populations [17]. Additional research is warranted to identify potential factors responsible for variable shedding of canine -associated genetic markers across different populations.

Other notable observations from reference pollution source testing include a higher prevalence of CowM3 compared to CowM2, likely due to local feeding practices [49,50]. A low sensitivity of GFD to avian sources (15.3%) was also observed suggesting that trends observed in Tillamook Bay Watershed samples are likely an underestimate of the true impact of birds on water quality. Additional research is needed to identify improved bird-associated genetic markers with a broader host distribution. In summary, performance evaluation of fecal source identification methods based on local reference pollution sources provides crucial information necessary for proper interpretation of qPCR findings, as well as an invaluable approach to select the most effective water quality management ‘tool box’.

#### The censored data challenge

Measurements whose values are known only to be above or below a defined threshold are called ‘censored’ data. For qPCR fecal source identification applications, a censored data situation occurs when samples yield Cq values greater than the respective LLOQ threshold (typically ranging from 35 to 39 Cq). This creates a censor data challenge in the sense that the true number of DNA target molecules in the sample cannot be firmly established. For fecal source identification qPCR data interpretation, these values are often deleted [51,52] or they are assigned an arbitrary fraction of the LLOQ [53,54] leaving conclusions vulnerable to claims of bias. In practice, a Cq value greater than the respective LLOQ including non-detects (Cq = 40) can represent a range of possible DNA target molecule concentrations corresponding to the complete absence of the target to the respective LLOQ (typically 8 to 10 copies/reaction). In this study, mean Cq values less than the respective LLOQ were observed in 88.4% of high-quality sample test reactions (8 qPCR assays · 586 samples/qPCR assay = 4,688 total number of mean Cq values; note that 12 samples failed qPCR data acceptance metrics) ranging from 45.7% (Rum2Bac) to 99.7% (DG37). This high proportion of censored data is a common occurrence in fecal source identification studies, but still remains a valuable source of information. There are many statistical analysis options available to properly incorporate censored data into estimates, hypotheses tests, and regressions without the need to ignore or substitute with fabricated numbers [55]. Additional research is warranted to further explore these options for data interpretation to help minimize bias and maximize fecal pollution trend insights.

## Conclusions

The objectives of this study were to implement the HF183/BacR287 and HumM2 human-associated qPCR standardized laboratory and data acceptance procedures in a large-scale field study and elucidate fecal pollution dynamics in host-associated genetic markers measured by qPCR across the Tillamook Bay Watershed. Intensive fecal source characterization of more than 600 samples collected from 29 sites over a year clearly demonstrates the feasibility and value of using standardized laboratory procedures and data acceptance metrics, especially for the HF183/BacR287 and HumM2 human-associated qPCR methods. Host-associated qPCR testing successfully uncovered numerous fecal pollution trends in the Tillamook Bay Watershed offering a multitude of new information to help local authorities improve water quality management. In addition, this large-scale field demonstration revealed key issues regarding qPCR data interpretation such as the importance of confirming method performance with local reference fecal samples and utilizing appropriate censored data analysis strategies. Further investigation of this rich data set will likely lead to additional water quality management information. Finally, it will be vital to continue to conduct large-scale, intensive field studies, such as the study presented here, in other water quality management arenas such as urban stormwater scenarios and recreational beach settings to tailor qPCR data interpretation strategies for these different applications.

## Supporting information

S1 Fig Histogram showing frequency of single-day maximum *E*. *coli* exceedance (> 406 MPN/100 mL) by sampling site over study period. (TIF)

S2 Fig Daily weather conditions recorded over the sampling period. Air temperature (°C; top), solar irradiance (kW‧hr/m^2^; middle), and 120-h precipitation (mm; bottom) are shown. Black diamonds indicate sampling event time points.
(TIF)

S3 Fig Estimated log_10_ copies per ng of total DNA for target and non-target reference fecal pollution sources. (TIF)

S1 Table Sampling site historical *E*. *coli* data analysis. Sampling site information and historical trends in single-day maximum *E*. *coli* exceedance across study area.
(PDF)

S2 Table Summary information for fecal source identification qPCR assays used in study. (PDF)

S3 Table Calibration model performance metrics for fecal source identification qPCR assays. (PDF)

S4 Table Site rankings based on average log_10_ concentrations of *E*. *coli* and eligible fecal source identification qPCR assays. (PDF)

S5 Table Land use data and sampling site average concentrations for eligible fecal pollution water quality measurements. (PDF)
