# Supplementary figures and images for: Large-scale implementation of standardized quantitative real-time PCR fecal source identification procedures in the Tillamook Bay Watershed
Source: PLoS One. 2019 Jun 6;14(6):e0216827. doi: 10.1371/journal.pone.0216827 (PMC6553688; doi:10.1371/journal.pone.0216827)

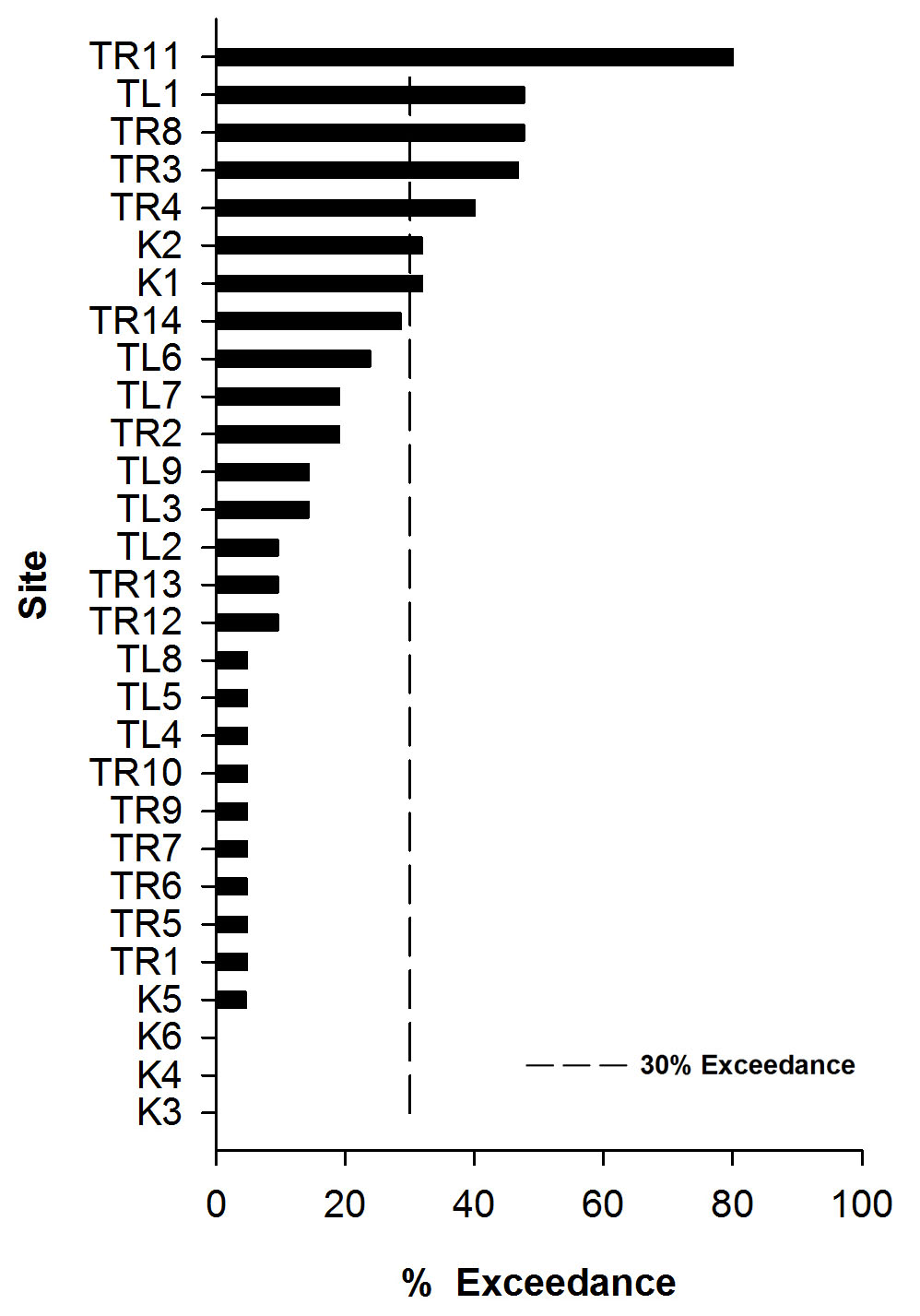

Supplement: S1 Fig — (TIF) [file pone.0216827.s001.tif]

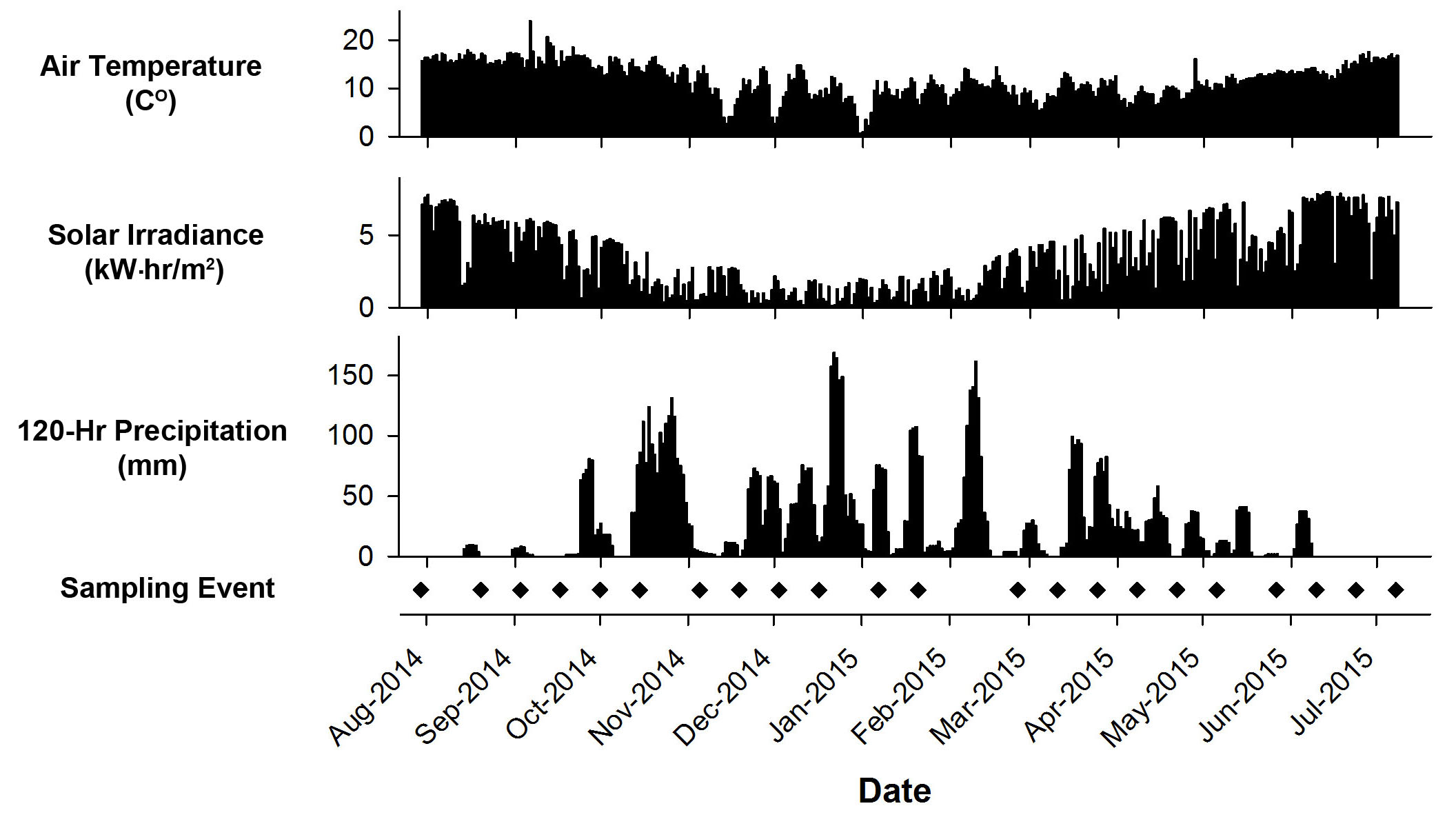

Supplement: S2 Fig — Air temperature (°C; top), solar irradiance (kW‧hr/m2; middle), and 120-h precipitation (mm; bottom) are shown. Black diamonds indicate sampling event time points. (TIF) [file pone.0216827.s002.tif]

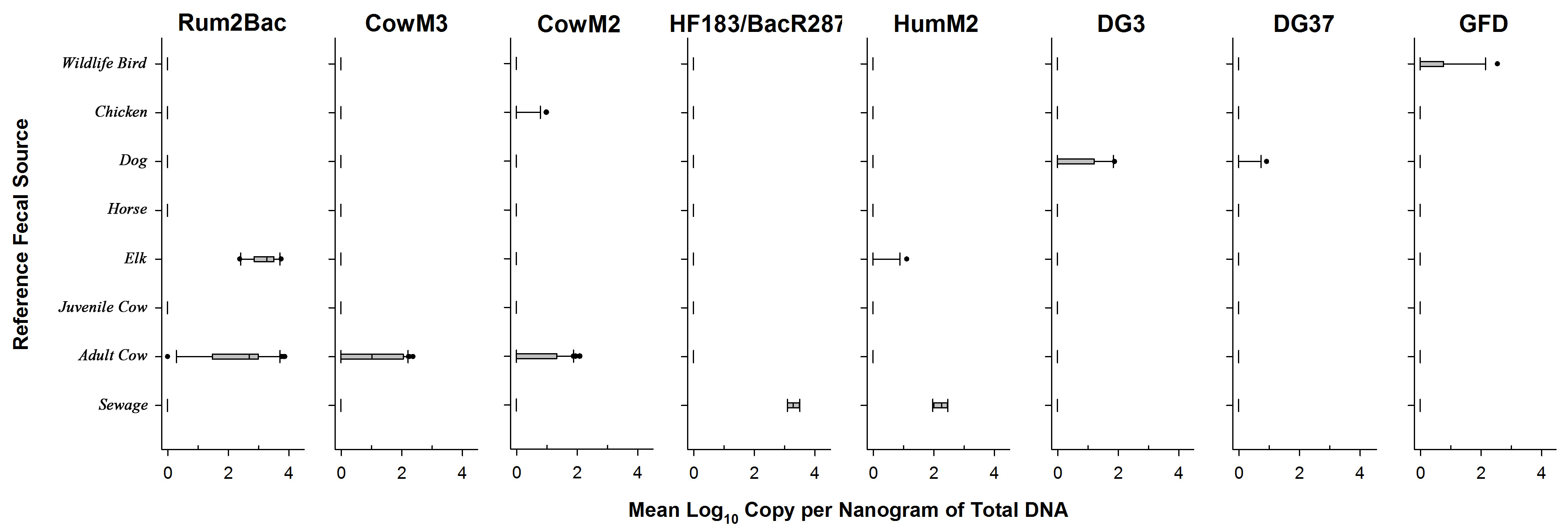

Supplement: S3 Fig — (TIF) [file pone.0216827.s003.tif]
