# Supplementary material for: Large-scale implementation of standardized quantitative real-time PCR fecal source identification procedures in the Tillamook Bay Watershed
Source: PLoS One. 2019 Jun 6;14(6):e0216827. doi: 10.1371/journal.pone.0216827 (PMC6553688; doi:10.1371/journal.pone.0216827)
Supplement: S1 Table — Sampling site information and historical trends in single-day maximum E. coli exceedance across study area. (PDF) [file pone.0216827.s004.pdf]

# Sampling Site Historical *E. coli* Data Analysis

**S1 Table.** Sampling site information and historical trends in single-day maximum *E. coli* exceedance across study area.

| River Tributary | Site | Historical *E. coli* ≥ 406 MPN/100mL | | | |
|---|---|---|---|---|---|
| | | n | % ± SE | Trend | p-value |
| Kilchis | K1 | 230 | 28.3 ± 3.0 | | |
| | K2 | 227 | 34.4 ± 3.2 | ↓ | 0.01 |
| | K3 | 229 | 0.4 ± 0.4 | | |
| | K4 | 234 | 0.4 ± 0.4 | | |
| | K5 | 234 | 2.1 ± 0.9 | | |
| | K6 | 229 | 1.3 ± 0.8 | | |
| Trask | TR1 | 234 | 12.4 ± 2.2 | | |
| | TR2 | 225 | 69.3 ± 3.1 | ↓ | < 0.0001 |
| | TR3 | 172 | 61.6 ± 3.7 | | |
| | TR4 | 175 | 58.3 ± 3.7 | | |
| | TR5 | 237 | 0.8 ± 0.6 | | |
| | TR6 | 237 | 3.4 ± 1.2 | | |
| | TR7 | 237 | 5.1 ± 1.4 | | |
| | TR8 | 237 | 59.5 ± 3.2 | ↓ | < 0.0001 |
| | TR9 | 237 | 7.2 ± 1.7 | | |
| | TR10 | 237 | 8.0 ± 1.8 | | |
| | TR11 | 236 | 77.5 ± 2.7 | | |
| | TR12 | 236 | 11.4 ± 2.1 | | |
| | TR13 | 237 | 15.2 ± 2.3 | | |
| | TR14 | 237 | 28.3 ± 2.9 | | |
| Tillamook | TL1 | 230 | 37.0 ± 3.2 | | |
| | TL2 | 231 | 5.2 ± 1.5 | | |
| | TL3 | 231 | 14.3 ± 2.3 | ↓ | 0.03 |
| | TL4 | 231 | 13.0 ± 2.2 | | |
| | TL5 | 231 | 11.7 ± 2.1 | | |
| | TL6 | 231 | 27.3 ± 2.9 | ↓ | < 0.0001 |
| | TL7 | 231 | 26.4 ± 2.9 | ↓ | < 0.0001 |
| | TL8 | 231 | 16.0 ± 2.4 | | |
| | TL9 | 231 | 12.6 ± 2.2 | | |

'n' denotes number of samples included in analysis.
'% ± SE' indicates percent exceeding 406 MPN 100/mL threshold with standard error between 2005-2015.
↓ indicates a significant decrease in % ± SE.
Trend represents sites where there was a significant temporal trend in probability of exceedance of the 406 MPN/100mL threshold ($p \leq 0.01$), after accounting for differences between wet and dry seasons.

Historical *E. coli* data from recommended sites were obtained from the Oregon Department of Environmental Quality. Sample site trends in historical *E. coli* single-day maximum exceedance probabilities (406 MPN/100mL) were based on 10 years of routine monitoring by local water quality managers summarized in Table S1. Percent exceedances ranged from consistently acceptable water quality 0.4 ± 0.4% (K4, K3) to chronically poor 77.5 ± 2.7% (TR11). A

significant ($p \leq 0.01$) decrease in the probability of an *E. coli* single-day maximum exceedance was observed for the K2, TR2, TR8, TL3, TL6, and TL7 sites (Table S1). All remaining sites showed no significant increase or decrease ($p > 0.01$) in water quality exceedances. Temporal trends in *E. coli* exceedance probabilities were calculated using a Firth's logistic regression approach.
