# Supplementary material for: Large-scale implementation of standardized quantitative real-time PCR fecal source identification procedures in the Tillamook Bay Watershed
Source: PLoS One. 2019 Jun 6;14(6):e0216827. doi: 10.1371/journal.pone.0216827 (PMC6553688; doi:10.1371/journal.pone.0216827)
Supplement: S2 Table — (PDF) [file pone.0216827.s005.pdf]

S2 Table. Summary information for fecal source identification qPCR assays used in study

Pollution Source	Assay	Chemistry	Annealing Temperature	Amplicon Length, bp	Reference
Human	HF183/BacR287	TaqMan	60°C	104	(1, 2)
	HumM2			101	(3, 4)
Ruminant	Rum2Bac			92	(5)
	CowM2			122	(6)
	CowM3			99	
Dog	DG3			285	(7)
	DG37			150	
Avian	GFD	SYBR	57°C	123	(8)

References

1. Green HC, Haugland R, Varma M, Millen HT, Borchardt MA, FIeld KG, et al. Improved HF183 quantitative real-time PCR assay for characterization of human fecal pollution in ambient surface water samples. Applied and Environmental Microbiology. 2014;80(10):3086-94.
2. USEPA. Method 1696: Characterization of human fecal pollution in water by HF183/BacR287 TaqMan quantitative polymerase chain reaction (qPCR) assay. Washington DC: United States Environmental Protection Agency, 2019 Contract No.: EPA 821-R-19-002.
3. Shanks OC, Kelty CA, Sivaganesan M, Varma M, Haugland RA. Quantitative PCR for genetic markers of human fecal pollution. Applied and Environmental Microbiology. 2009;75:5507-13.
4. USEPA. Method 1697: Characterization of human fecal pollution in water by HumM2 TaqMan quantitative polymerase chain reaction (qPCR) assay. Washington DC: United States Environmental Protection Agency, 2019 Contract No.: EPA 821-R-19-003.
5. Mieszkin S, Yala JF, Joubrel R, Gourmelon M. Phylogenetic analysis of *Bacteroidales* 16S rRNA gene sequences from human and animal effluents and assessment of rumaint faecal pollution by real-time PCR. Journal of Applied Microbiology. 2010;108:974-84.
6. Shanks OC, Atikovic E, Blackwood AD, Lu J, Noble RT, Santo Domingo J, et al. Quantitative PCR for Detection and Enumeration of Genetic Markers of Bovine Fecal Pollution. Applied and Environmental Microbiology. 2008;74(3):745-52.
7. Green HC, White KM, Kelty CA, Shanks OC. Development of rapid canine fecal source identification PCR-based assays. Environmental Science and Technology. 2014.
8. Green HC, Dick LK, Gilpin B, Samadpour M, Field KG. Genetic markers for rapid PCR-based identificationof gull, Canada goose, duck, and chicken fecal contamination in water. Applied and Environmental Microbiology. 2012;78:503-10.
