# Supplementary material for: Large-scale implementation of standardized quantitative real-time PCR fecal source identification procedures in the Tillamook Bay Watershed
Source: PLoS One. 2019 Jun 6;14(6):e0216827. doi: 10.1371/journal.pone.0216827 (PMC6553688; doi:10.1371/journal.pone.0216827)
Supplement: S3 Table — (PDF) [file pone.0216827.s006.pdf]

S3 Table. Calibration model performance metrics for fecal source identification qPCR assays.

Assay	Slope	Y-Intercept	*E*	R^2	LLOQ	IAC Thresholds	
						Interference	*Competition*
CowM2	-3.49 ± 0.05	41.52 ± 0.18	0.93	0.985	38.34	-	-
CowM3	-3.51 ± 0.03	39.77 ± 0.25	0.93	0.983	36.75	-	-
Rum2Bac	-3.48 ± 0.03	41.17 ± 0.13	0.94	0.991	37.94	-	-
DG3	-3.49 ± 0.04	38.10 ± 0.13	0.93	0.989	34.83	-	-
DG37	-3.49 ± 0.04	38.62 ± 0.22	0.93	0.990	35.62	-	-
GFD	-3.58 ± 0.04	40.48 ± 0.13	0.90	0.981	37.11	-	-
HF183/BacR287	-3.40 ± 0.04	38.15 ± 0.22	0.97	0.984	35.18	32.8-37.8	27.9
HumM2	-3.44 ± 0.04	40.47 ± 0.19	0.95	0.987	37.40	34.4-38.6	30.1

E denotes amplification efficiency ($E = 10^{(-1/slope)} - 1$).
R^2 indicates calibration model linearity.
LLOQ represents the lower limit of quantification.
IAC denotes internal amplification control.
