# Supplementary material for: Large-scale implementation of standardized quantitative real-time PCR fecal source identification procedures in the Tillamook Bay Watershed
Source: PLoS One. 2019 Jun 6;14(6):e0216827. doi: 10.1371/journal.pone.0216827 (PMC6553688; doi:10.1371/journal.pone.0216827)
Supplement: S4 Table — (PDF) [file pone.0216827.s007.pdf]

S4 Table. Site rankings based on average concentrations of *E. coli* (log_{10} MPN/100 mL) and eligible fecal source identification qPCR assays (log_{10} copies per reaction).

Site	% *E. coli* exceedance	*E. coli*		Rum2Bac		Cowm2		Cowm3		GFD		HF183/BacR287	
		Score	*Rank*	*Score*	*Rank*	*Score*	*Rank*	*Score*	*Rank*	*Score*	*Rank*	*Score*	*Rank*
TR11	80.00	2.76	1	-0.04	nr	-1.08	nr	-0.47	nr	0.04	9	0.14	6
TL1	47.62	2.52	2	1.79	5	-0.15	nr	-0.27	nr	0.07	8	-0.26	nr
TR3	46.67	2.46	3	2.12	1	0.35	1	1.02	1	0.46	2	0.22	4
TR8	47.62	2.39	4	1.58	6	-0.04	nr	0.33	3	0.31	4	-0.34	nr
TR4	40.00	2.38	5	1.86	2	-0.20	nr	0.50	2	0.19	7	0.44	2
TL7	19.05	2.22	6	1.86	3	-0.43	nr	-0.26	nr	0.24	6	-0.28	nr
T14	28.57	2.21	7	0.79	13	-0.89	nr	-0.41	nr	-0.10	nr	-0.11	nr
K2	31.82	2.19	8	0.85	12	-0.83	nr	-0.68	nr	0.49	1	-0.39	nr
TR2	19.05	2.16	9	0.75	14	-0.47	nr	0.15	4	0.02	10	0.45	1
TL6	23.81	2.14	10	1.86	4	-0.41	nr	-0.22	nr	-0.13	nr	0.15	5
K1	31.82	2.08	11	-0.43	nr	-1.55	nr	-1.55	nr	0.30	5	-0.61	nr
TL9	14.29	2.04	12	0.37	20	-1.10	nr	-1.55	nr	-0.16	nr	0.09	7
TL5	4.76	2.01	13	1.37	7	-0.85	nr	-0.49	nr	-0.25	nr	0.25	3
TL2	9.52	1.93	14	0.60	17	-1.53	nr	-1.08	nr	-0.30	nr	-0.40	nr
TR13	9.52	1.90	15	0.29	21	-0.57	nr	-0.71	nr	-0.29	nr	-0.70	nr
TL4	4.76	1.88	16	1.13	8	-1.36	nr	-0.42	nr	-0.17	nr	-0.29	nr
TR3	4.76	1.87	17	0.74	15	-1.10	nr	-0.61	nr	-0.41	nr	-0.18	nr
TR7	4.76	1.85	18	0.40	19	-1.57	nr	-0.89	nr	-0.42	nr	-0.89	nr
TR9	4.76	1.81	19	0.92	10	-1.10	nr	-0.41	nr	-0.48	nr	-0.71	nr
TL3	14.29	1.77	20	1.12	9	-0.59	nr	-0.60	nr	-0.20	nr	-0.60	nr
TL8	4.76	1.77	21	0.90	11	-0.85	nr	-0.40	nr	0.39	3	-0.38	nr
TR6	4.76	1.71	22	0.63	16	-1.57	nr	-1.12	nr	-0.39	nr	-1.12	nr
TR1	4.76	1.68	23	-0.06	nr	-1.00	nr	-0.50	nr	-0.37	nr	-0.43	nr
K5	4.55	1.62	24	0.58	18	-1.57	nr	-0.89	nr	-0.49	nr	-0.56	nr
K6	0.00	1.61	25	-0.11	nr	-1.57	nr	-1.12	nr	-0.42	nr	-0.89	nr
TR5	4.76	1.57	26	0.07	22	-1.57	nr	-1.55	nr	-0.44	nr	-0.87	nr
TR12	9.52	1.54	27	-1.10	nr	-1.55	nr	-1.55	nr	-0.32	nr	-0.44	nr
K4	0.00	1.47	28	-0.16	nr	-1.55	nr	-0.89	nr	-0.89	nr	-1.12	nr
K3	0.00	1.38	29	-0.01	nr	-0.89	nr	-1.55	nr	-0.61	nr	-1.10	nr

‘nr’ denotes not ranked.
