# Supplementary material for: Large-scale implementation of standardized quantitative real-time PCR fecal source identification procedures in the Tillamook Bay Watershed
Source: PLoS One. 2019 Jun 6;14(6):e0216827. doi: 10.1371/journal.pone.0216827 (PMC6553688; doi:10.1371/journal.pone.0216827)
Supplement: S5 Table — (PDF) [file pone.0216827.s008.pdf]

S5 Table. Land use data and sampling site average concentrations for eligible fecal pollution water quality measurements.

River Tributary	Site	Land Use Parameters				Eligible Water Quality Metrics*			
		Human Population Count (log_{10})	*Percent Non-Sewer Area*	*Permitted Cattle Count (log_{10})*	*Percent Cropland*	*E. coli*	*Rum2Bac*	*HF183/BacR287*	*GFD*
Kilchis	K1	2.32	2.24	0.00	0.64	2.08	-0.43	-0.61	0.30
	K6	0.27	0.21	0.00	25.73	1.61	-0.11	-0.89	-0.42
	K2	1.81	3.76	3.39	3.67	2.19	0.85	-0.39	0.49
	K5	2.19	0.02	2.94	0.18	1.62	0.58	-0.56	-0.49
	K4	1.92	0.01	0.00	0.13	1.47	-0.16	-1.12	-0.89
	K3	1.81	0.01	0.00	0.09	1.38	-0.01	-1.10	-0.61
Tillamook	TL8	3.14	0.88	3.71	1.01	1.77	0.90	-0.38	0.39
	TL7	3.04	0.22	3.46	0.84	2.22	1.86	-0.28	0.24
	TL4	2.85	0.11	2.92	0.57	1.88	1.13	-0.29	-0.17
	TL3	2.84	0.11	2.92	0.57	1.77	1.12	-0.60	-0.20
	TL2	2.76	0.10	2.83	0.50	1.93	0.60	-0.40	-0.30
	TL9	1.89	0.22	0.00	0.53	2.04	0.37	0.09	-0.16
	TL6	2.93	0.15	3.28	0.85	2.14	1.86	0.15	-0.13
	TL5	2.86	0.11	2.92	0.57	2.01	1.37	0.25	-0.25
	TL1	1.04	0.01	2.30	0.31	2.52	1.79	-0.26	0.07
Trask	TR3	2.83	4.51	3.34	0.08	2.46	2.12	0.22	0.46
	TR4	3.27	4.63	3.34	0.40	2.38	1.86	0.44	0.19
	TR2	2.93	7.44	3.74	7.29	2.16	0.75	0.45	0.02
	TR1	2.38	2.62	3.24	6.30	1.68	-0.06	-0.43	-0.37
	TR13	2.53	0.88	2.71	2.00	1.90	0.29	-0.70	-0.29
	TR12	1.91	0.69	0.00	0.61	1.54	-1.10	-0.44	-0.32
	TR11	1.75	0.34	0.00	0.22	2.76	-0.04	0.14	0.04
	TR5	2.21	0.01	0.00	0.09	1.57	0.07	-0.87	-0.44
	TR10	3.81	0.18	4.09	0.40	1.87	0.74	-0.18	-0.41
	TR6	2.73	0.03	3.25	0.20	1.71	0.63	-1.12	-0.39
	TR14	2.55	0.86	2.71	2.16	2.21	0.79	-0.11	-0.10
	TR7	3.08	0.09	3.71	0.29	1.85	0.40	-0.89	-0.42
	TR8	3.67	0.18	4.01	0.38	2.39	1.58	-0.34	0.31
	TR9	3.80	0.18	4.06	0.40	1.81	0.92	-0.71	-0.48

* Indicates 20% or more site averages > 0.
Human population estimated using EnviroAtlas Dasymetric toolbox (9).
Maximum permitted CAFO cattle population and percent non-sewered area estimated using SPARROW data layers (10).
Percent cropland was estimated from the National Gap Analysis Project Land Cover data set (https://gapanalysis.usgs.gov/gaplandcover/).
E. coli reported as MPN per 100 mL.
Rum2Bac, HF183/BacR287, and GFD qPCR values reported as log_{10} copies per reaction.
